# Germline-restricted chromosomes of the songbirds

**DOI:** 10.18699/VJGB-23-75

**Published:** 2023-10

**Authors:** P.M. Borodin

**Affiliations:** Institute of Cytology and Genetics of the Siberian Branch of the Russian Academy of Sciences, Novosibirsk, Russia

**Keywords:** germline-restricted chromosomes, avian genome evolution, programmed DNA elimination, хромосомы, ограниченные зародышевой линией, эволюция генома птиц, запрограммированная элиминация ДНК

## Abstract

Germline-restricted chromosomes (GRCs) are present in the genomes of germline cells and absent from somatic cells. A GRC is found in all species of the songbirds (Passeri) and in none of the other bird orders studied to date. This indicates that GRC originated in the common ancestor of the songbirds. The germline-restricted chromosome is permanently absent from somatic cells of the songbird, while female germline cells usually contain two copies of GRC and male ones have one copy. In females, GRCs undergo synapsis and restricted recombination in their terminal regions during meiotic prophase. In males, it is almost always eliminated from spermatocytes. Thus, GRC is inherited almost exclusively through the maternal lineage. The germline-restricted chromosome is a necessary genomic element in the germline cells of songbirds. To date, the GRC genetic composition has been studied in four species only. Some GRC genes are actively expressed in female and male gonads, controlling the development of germline cells and synthesis of the proteins involved in the organization of meiotic chromosomes. Songbird species vary in GRC size and genetic composition. The GRC of each bird species consists of amplified and modified copies of genes from the basic genome of that species. The level of homology between GRCs of different species is relatively low, indicating a high rate of genetic evolution of this chromosome. Transmission through the maternal lineage and suppression of the recombination contribute significantly to the accelerated evolution of GRCs. One may suggest that the rapid coordinated evolution between the GRC genes and the genes of the basic genome in the songbirds might be responsible for the explosive speciation and adaptive radiation of this most species-rich and diverse infraorder of birds.

## Abstract (ru)

Хромосомы, ограниченные зародышевой линией (germline-restricted chromosomes – GRC), присутствуют в геномах герминативных клеток и запрограммированно элиминируются из соматических клеток в ходе развития. Они крайне редко возникают в ходе эволюции. Еще реже они становятся необходимыми элементами геномов герминативных клеток крупных таксонов. Такая хромосома, ограниченная зародышевой линией, была обнаружена у всех исследованных представителей подотряда воробьинообразных певчих птиц. Ни у одного из представителей других отрядов птиц GRC не выявлено. Это свидетельствует о том, что GRC возникла у общего предка воробьинообразных певчих птиц. У всех представителей этого подотряда GRC присутствует, как правило, в двух копиях в герминативных клетках самок и в одной копии у самцов и отсутствует в соматических клетках. У самок GRC синаптируют и рекомбинируют в профазе мейоза. У самцов они почти всегда элиминируются из сперматоцитов. Таким образом, GRC наследуется почти исключительно по материнской линии. Хромосомы, ограниченные зародышевой линией, – необходимый элемент генома герминативных клеток певчих птиц. На сегодняшний день исследованы геномы GRC четырех видов. Некоторые гены GRC активно экспрессируются в гонадах самцов и самок, контролируют развитие герминативных клеток, синтез белков, вовлеченных в организацию мейотических хромосом. GRC разных видов различаются по размерам и генетическому составу. Геном GRC каждого вида птиц состоит из амплифицированных перестроенных копий генов основного генома данного вида. Уровень гомологии между GRC разных видов довольно низок. Это указывает на высокую скорость эволюции генетического состава хромосомы. Значительный вклад в ускорение эволюции GRC вносят передача этой хромосомы по материнской линии и подавление рекомбинации в ней. Можно считать, что быстрая согласованная эволюция генов GRC и генов основного набора воробьинообразных певчих птиц играет важную роль в видообразовании и адаптивной радиации представителей этого самого богатого видами и разнообразного подотряда птиц.

## Programmed DNA elimination

In the late 19th century, A. Weismann proposed one of the most influential ideas in modern biology. He suggested a fundamental distinction between somatic cells and germ line cells (Weismann, 1890, 1893). According to A. Weismann, germline cells possess a complete set of hereditary material (germ plasm), while somatic cells undergo unequal divisions during their differentiation and specialization, which leads to a fragmentation of the germ plasm, with different daughter somatic cells acquiring its different fragments.

The former part of Weismann’s hypothesis became the basis of modern evolutionary biology, genetics, and developmental biology. The latter part underwent significant modifications. Unequal division of somatic cells during early development in the vast majority of modern multicellular organisms does not lead to genome fragmentation (as suggested by Weismann), but rather to the uneven distribution of its products (proteins and RNAs) among the daughter cells. This, in turn, leads to progressive differentiation of their gene activity and, ultimately, to their specialization (Dröscher, 2014).

However, somatic cells in some species behave according to Weismann’s hypothesis: they lose a portion of their genomes during the differentiation. This phenomenon was previously referred to as “chromatin diminution”. The prevailing term now is “programmed DNA elimination” (Wang, Davis, 2014). The first example of chromatin diminution was discovered by T. Boveri in the nematode Ascaris megalocephala (Boveri, 1887). During the early stages of its development, the nuclei of certain cells progressively lose a portion of their chromosomal material. T. Boveri suggested that chromatin diminution is part of a normal developmental program necessary for the formation of specific tissues and organs in the worm (Maderspacher, 2008).

Programmed DNA elimination is observed in various taxa of multicellular organisms. It plays a key role in regulating gene expression and maintaining genomic stability. Various molecular pathways involved in selective DNA removal during chromatin diminution have been identified (Wang, Davis, 2014; Smith, 2018; Smith et al., 2021; Dedukh, Krasikova, 2022). It has been shown that the regions of the genome that are eliminated are the homologous counterparts of those undergoing silencing in other multicellular organisms, such as oncogenes and other early developmental genes (Wang et al., 2012; Streit et al., 2016; Smith, 2018; Smith et al., 2018).

Thus, programmed DNA elimination can be considered a radical form of silencing, a specific way to resolve the problem of antagonistic pleiotropy between early and late genetic programs. Apparently, this mechanism of developmental regulation through programmed DNA elimination has emerged repeatedly in evolution and has proven to be an evolutionary dead end. Its erratic distribution across the phylogenetic tree of multicellular animals supports this hypothesis (Smith et al., 2021).

## Whole chromosome elimination

Elimination in somatic cells usually affects chromosome fragments rather than entire chromosomes. Cases of whole chromosome elimination are less frequent (Dedukh, Krasikova, 2022). In several species of mammals and invertebrates from different taxa, chromosome elimination serves as a mechanism of sex determination and/or dosage compensation of genes on heterogametic sex chromosomes (Watson et al., 1998; Wang, Davis, 2014; Smith et al., 2021).

Conflict between host genomes and their genomic parasites sometimes leads to the emergence of additional (i. e., nonessential for survival) chromosomes. They are called B-chromosomes, in contrast to A-chromosomes of the basic karyotype. B-chromosomes vary in size, morphology, and genetic content between species, among individuals within a species, and even among individual cells of an organism (Houben et al., 2014; Camacho, 2022). Several studies detected a tendency for B-chromosomes to accumulate in germ cells and be lost in somatic cells (Camacho et al., 2000; Camacho, 2022).

B-chromosomes may not be present in all populations of a particular species. Species with B-chromosomes also exhibit erratic phylogenetic distribution (D’Ambrosio et al., 2017). For example, B-chromosomes are present in only 16 species from 11 rather species-rich genera out of 1130 species described in the “Atlas of Mammalian Chromosomes” (Graphodatsky et al., 2020). This suggests that B-chromosomes are typical genomic parasites that occasionally arise in some species, sometimes gain pyrrhic victories and spread in populations, but ultimately either become completely eliminated from the genomes of these species or go extinct along with their hosts (Camacho et al., 2000; Houben et al., 2014; Houben, 2017; Camacho, 2022; Chen et al., 2022).

In some cases, however, such parasitic chromosomes may achieve evolutionary success and become an indispensable part of the genome in germ cells of all or most species within larger taxa. Such germline-restricted chromosomes (GRCs) have been discovered in almost all studied species from three dipteran families: fungus gnats (Sciaridae), gall midges (Cecidomyiidae), and non-biting midges (Chironomidae) (Hodson, Ross, 2021), as well as in all studied species of passerine birds (Passeriformes) – the most species-rich and diverse order of birds (Torgasheva et al., 2019; Borodin et al., 2022).

A comprehensive review of current knowledge on GRCs in Diptera was provided by C.N. Hodson and L. Ross (2021). A recent review of GRCs in passerines was published by a group of authors, including nearly all European researchers of this chromosome and the author of this article (Borodin et al., 2022), in a special issue dedicated to non-Mendelian inheritance and meiotic drive (Hanlon, Larracuente, 2022). It paid special attention to hypotheses regarding the mechanisms of GRC transmission in the germline and its elimination from somatic cells. Another review analyzing GRC in the context of genomic conflicts appeared online in September 2023 (Vontzou et al., 2023).

This review will address issues related to the structure, function, and evolution of this enigmatic chromosome.

## Discovery of GRC in birds

GRC in birds was first identified by Argentine cytogeneticists M.I. Pigozzi and A.J. Solari in the zebra finch (Taeniopygia guttata) (Pigozzi, Solari, 1998). They stumbled upon it entirely by chance during a comparative study of chromosome synapsis and recombination in bird meiosis. Among the numerous bird species they examined (Solari, Moses, 1973; Rahn, Solari, 1986; Solari, 1992; Solari, Pigozzi, 1993; Pigozzi, Solari, 1997), the zebra finch was the first representative of Passeriformes. In the meiotic cells of this species, they observed something that had not been seen in any previously studied species.

The germline cells of both male and female zebra finches contained an additional chromosome that was not present in bone marrow and other somatic cells. This additional chromosome was larger than all other chromosomes. It was euchromatic in oocytes and heterochromatic in spermatocytes (Pigozzi, Solari, 1998). Furthermore, in female germline cells, it was usually present in two copies, whereas in males, it was present in a single copy (Pigozzi, Solari, 2005).

Another unexpected feature of this chromosome was its absence not only in somatic cells but also in spermatozoa. During pachytene, diplotene, and metaphase I of meiosis, the GRC was localized at the periphery of chromosomal plates. However, it was absent in metaphase II of male meiosis. Additionally, round dense DAPI-positive bodies were observed adjacent to some spermatocytes. Electron microscopy revealed that these round bodies were surrounded by a double membrane. M.I. Pigozzi and her colleagues hypothesized that these micronuclei contained GRCs eliminated after the first meiotic division (Pigozzi, Solari, 1998, 2005; Itoh et al., 2009; Goday, Pigozzi, 2010; Schoenmakers et al., 2010).

Subsequently, A.A. Torgasheva et al. (2019) confirmed this hypothesis in a direct experiment. They microdissected these micronuclei, prepared DNA probes from them, and hybridized these probes to preparations of pachytene spermatocytes and oocytes. In all cases, they observed a strong, specific hybridization signal on the GRCs (Torgasheva et al., 2019). Since the GRC was absent from spermatozoa, M.I. Pigozzi and A.J. Solari (2005) and Y. Itoh et al. (2009) suggested that it must be inherited exclusively through the maternal lineage.

Initially, M.I. Pigozzi and A.J. Solari (1998) classified the zebra finch GRC as a B-chromosome. However, they noted its unique characteristics. B-chromosomes usually vary in number in somatic cells and tend to accumulate in germline cells. The zebra finch GRC was absent in somatic cells and always present in every germline cell. From the outset of the GRC investigation, it became clear that this chromosome was not a facultative but an obligate element of the germline cell genome, essential for gametogenesis.

## GRC at the phylogenetic tree of birds

The zebra finch GRC has long been considered an intriguing genetic curiosity, the sole instance of the B-chromosome in birds. The discovery of GRC in germline cells of the Bengalese finch (Lonchura striata domestica) (del Priore, Pigozzi, 2014) did not alter this opinion. However, the situation changed dramatically in 2018–2019, when three independent research groups published data indicating the presence of GRC in the germline of many species of birds (Biederman et al., 2018; Kinsella et al., 2019; Torgasheva et al., 2019).

**Fig. 1.**
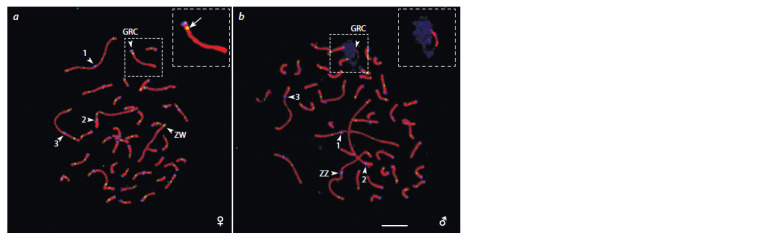
Germline cells of the great tit at the pachytene stage after immunostaining with antibodies against SYCP3 (red), centromeric proteins (blue), and MLH1 (green). a – oocyte containing a GRC bivalent; b – spermatocyte with a GRC univalent. The arrowheads indicate the centromeres of the GRC, the three largest macrobivalents, and the heteromorphic ZW bivalent with misaligned centromeres. The arrow points to the MLH1 signal in the pericentromeric region of the GRC bivalent. Scale 5 μm. Photographs from the article (Torgasheva et al., 2019), published in “Proceedings of the National Academy of Sciences”, USA, under the CC-BY-SA 4.0 license (modified).

A.A. Torgasheva et al. (2019) obtained direct evidence of the antiquity of GRC in birds and its wide distribution among songbirds. They conducted cytogenetic screening of 15 songbird species and 8 bird species outside this suborder. They used immunolocalization of SYCP3, the major protein of the synaptonemal complex (axial scaffold of meiotic prophase chromosomes), centromeric proteins, and MLH1, the mismatch repair protein, which marks sites of homologous recombination (Fig. 1).

**Fig. 2.**
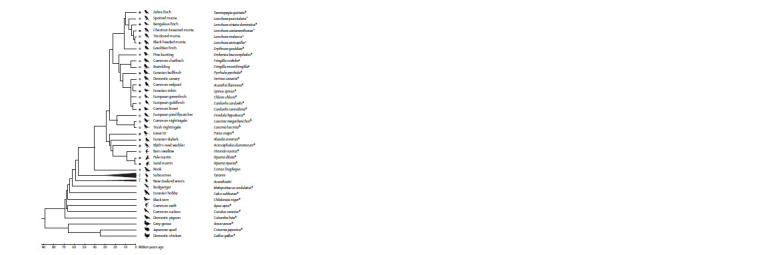
Phylogenetic tree of birds with investigated pachytene karyotypes. Black circles indicate species with macro-GRC, white circles indicate species with micro-GRC, and the absence of circles denotes the absence of GRC. Symbols represent data sources: * Torgasheva et al., (2019); # Malinovskaya et al., (2022); ^ Sotelo-Muñoz et al. (2022); & Poignet et al. (2021). The diagram is adapted from the article by P.M. Borodin et al. (2022) published in “Chromosome Research” under the CC-BY-SA 4.0 license. (modified).

A.A. Torgasheva et al. (2019) demonstrated that GRC is present in all pachytene cells of all investigated individuals of songbird species, including the rook (Corvus frugilegus), a representative of the Corvida infraorder, the sister group to the Passerida infraorder, which includes all other songbird families (Fig. 2). The size of GRC varies widely, with some species having macrochromosomal GRC of size rank 1 to 3, and others having micro-GRC. No phylogenetic clustering has been observed for this trait: closely related species differ greatly in the size of their GRC (see Fig. 2). However, GRC exhibits considerable conservatism in terms of its morphology; in nearly all investigated species, GRC is an acrocentric chromosome. The exception is the pied flycatcher, whose GRC is metacentric (Torgasheva et al., 2019; Malinovskaya et al., 2022).

GRC has not been detected in any bird species outside the order Passeriformes, including the budgerigar (Melopsittacus undulatus), a representative of the order Psittaciformes, which is the sister group to the order Passeriformes (Torgasheva et al., 2019). The list of species examined for the presence of GRC now includes 27 songbird species with GRC and 9 bird species without GRC outside this suborder (see Fig. 2) (Borodin et al., 2022).

Based on cytogenetic screening, A.A. Torgasheva et al. (2019) concluded that GRC is likely present in the germline cell genomes of all songbirds and absent in all other birds. They suggested that GRC originated in the common ancestor of the Passeri suborder, which includes approximately 5000 species. The germline cells of representatives of the Tyranni suborder, consisting of approximately 1200 species, and the Acanthisitti suborder, which includes two species of New Zealand wrens, have not been analyzed yet. Therefore, it is still unknown whether they have GRC. If they have it, this would indicate that GRC emerged much earlier, in the common ancestor of all passerine birds.

Independent evidence of the evolutionary antiquity of the GRC has been obtained by comparing the results of sequencing the generative and somatic tissues of the zebra finch (Kinsella et al., 2019) and subtractive transcriptomic analysis (Biederman et al., 2018). Over a hundred GRC-specific genes (gametologs) and their somatic paralogs (somatologs) have been identified on at least 19 A-chromosomes.

The results of phylogenetic analysis enabled the categorization of GRC-specific genes into five evolutionary strata based on the degree of divergence from their somatic paralogs (Kinsella et al., 2019).

The level of divergence between gametologs of the youngest stratum 5 and their somatic counterparts is comparable to the level of divergence among the subspecies of the zebra finch. Gametologs of stratum 4 emerged during the divergence of the zebra finch from the long-tailed finch (Poephila acuticauda) and the diamond firetail (Stizoptera bichenovii). Stratum 3 comprises gametologs that originated from a common ancestor of estrildid species (Estrildidae), stratum 2 corresponds to the ancestor of the Passerida infraorder. The most ancient stratum 1 arose in the common ancestor of the suborder Passeri.

Thus, both cytological and molecular genetic data unambiguously indicate the monophyletic origin of the GRC

## Genetic content of GRC

What does GRC contain that makes it an obligate element of the germline genome of all songbirds? At the time of writing this article, the results of genomic analysis of GRC have been published for only four species of songbirds: the zebra finch (Kinsella et al., 2019), two species of nightingales: western, Luscinia megarhynchos and eastern, L. luscinia (Schlebusch et al., 2023), and the Eurasian blue tit, Cyanistes caeruleus (Mueller et al., 2023).

The most extensively studied zebra finch GRC contains a set of genes crucial for the development of the reproductive system. Contrary to expectations, mobile elements and satellite DNA are not more abundant in GRC compared to A-chromosomes; in fact, they are less abundant (Kinsella et al., 2019; Torgasheva et al., 2019). Transcriptomic analysis revealed the transcription of at least six GRC-specific genes in the testes and 32 genes in the ovaries. Mass spectrometry analysis confirmed the translation of mRNA from some of these genes in the gonads of both sexes. Gene categories related to gonad formation in females and reproduction are overrepresented in the list of GRC-specific genes. Orthologs of many zebra finch GRC-specific genes are predominantly expressed in the chicken gonads of both sexes (Kinsella et al., 2019). Both the macro-GRC of the zebra finch and the micro-GRC of the blue tit contain a functional paralog of the BMP15 transcription factor, which plays an important role in follicle maturation in the ovaries of birds and other vertebrates (Mueller et al., 2023). In the GRC of the blue tit, J.C. Mueller et al. (2023) revealed the genes controlling the formation of the synaptonemal complex and other genes controlling chromosome synapsis and recombination.

Some of the gametologs found in the zebra finch GRC, including the most ancient ones (bicc1 and trim71), are subjected to purifying selection (Kinsella et al., 2019). The products of their orthologs play an important role in the differentiation of mammalian embryonic cells (Uhlén et al., 2015). The bicc1 gene encodes an RNA-binding protein that modulates protein translation during embryonic development. The product of the trim71 gene binds to microRNAs and supports the growth and proliferation of embryonic stem cells. It is also involved in cell cycle control. The gametolog of the puf60 gene shows signs of recent positive selection. It encodes a protein playing an important role in multiple nuclear processes, including pre-mRNA splicing and transcriptional regulation (Kinsella et al., 2019). These data clearly indicate the functional significance of at least some of the gametologs. However, some genes in the GRC of the blue tit and many genes in both species of nightingales have undergone pseudogenization and presumably lost their functional significance (Schlebusch et al., 2022; Mueller et al., 2023).

Interestingly, the duplication of genes from the somatic genome into GRC did not lead to the loss of their “original” copies. Moreover, in the blue tit, a positive correlation in the number of copies was found between gametologs and their somatologs (r = 0.35, P = 0.001) (Mueller et al., 2023).

Thus, GRCs of all four investigated species contain copies of A-chromosome genes, many of which control the development and functioning of germ cells.

## Sexual dimorphism in the number of GRCs in the germ cells and its behavior in the meiotic prophase

In almost all male pachytene cells (with rare exceptions, which I will discuss below), at least one GRC is present. It appears as a univalent with a twisted, sometimes fragmented, single axial element of the synaptonemal complex surrounded by chromatin, intensely labeled with anticentromere antibodies. The univalent does not undergo synapsis and recombination neither with itself nor with A-chromosomes. In almost all female pachytene cells (with rare exceptions, which I will discuss below), two GRCs are present. They synapse along their entire length, forming bivalents, and undergo recombination. The only difference between GRC bivalents and A-chromosome bivalents is the distribution of the recombination sites. There are fewer recombination cites on GRC bivalents compared to A-chromosome bivalents of similar size, and they occupy more distal positions on the GRC bivalents (Pigozzi, Solari, 1998, 2005; Torgasheva et al., 2019, 2021; Malinovskaya et al., 2020).

With an increase in the number of species and individuals studied, and the use of molecular probes, many exceptions were identified. Five females (one zebra finch and four sand martins) out of 50 individuals of seven species examined had one GRC (instead of two) in all their pachytene cells (Borodin et al., 2022). Four females of the great tit out of seven examined were mosaic for the number of GRCs: one GRC was observed in a small fraction of their cells (from 2 to 26 %), while the rest contained two GRCs (Torgasheva et al., 2021).

Among 76 males of 26 investigated species, no individuals with more than one GRC in all germ cells have been found so far. However, nine males (seven pale martins, one great tit, one pied flycatcher, and one black-headed munia) exhibited mosaicism in the number of GRCs (Borodin et al., 2022). Some germ cells of mosaic males contained two or even three GRCs (Malinovskaya et al., 2020). The male black-headed munia was mosaic not only in the number but also in the size of GRCs. Some of its cells contained one macro-GRC and one micro-GRC (Sotelo-Muñoz et al., 2022).

Sexual dimorphism in the meiotic behavior of GRC is maintained even in the germ cells, which contain atypical numbers of GRC for their respective sexes. The two axial elements of GRC in male pachytene cells show incomplete synapsis and extremely rare recombination (Malinovskaya et al., 2020). However, even in the case of such recombination occurring in male meiosis, it would not play a role in the evolution of the genetic composition of the GRC due to the extremely low chance of GRC transmission through males (Pei et al., 2022).

The almost exclusive transmission of the GRC through the maternal lineage, coupled with the fact that recombina- tion in most regions of the GRC is suppressed, suggests that the GRC in songbirds represents a new genomic element subject to the action of the Meller’s ratchet, in addition to the sufficiently gene-rich W chromosome and the mitochondrial genome. Non-recombining genomic elements are characterized by an exceptionally high rate of fixation of point and structural mutations (Gabriel et al., 1993). It can be hypothesized that the GRC should evolve at a high rate and accelerate the evolution of the entire suborder of songbirds.

## Evolution of the GRC

The rate of GRC evolution can be assessed by the degree of divergence in the genetic composition of the GRC among different bird species. A rough estimation of the degree of homology between GRCs of several bird species was obtained using reciprocal fluorescent in situ hybridization (FISH). This method revealed an astonishingly low degree of homology between GRCs of different species, which could indicate a rapid evolution of the genetic composition of the GRC (Torgasheva et al., 2019).

The initial results of the genomic analysis of GRCs from different species confirm the assumption of an exceptionally high rate of GRC evolution. The GRCs of the western nightingale (L. megarhynchos) and the common nightingale (L. luscinia), which have undergone independent evolution for only 1.8 million years, show considerable divergence. Among the 585 gametologs of the western nightingale and the 406 gametologs of the common nightingale, only 192 of them are shared. Among them, only 25 are shared with the GRC of the zebra finch. In other words, only one-third of the identified gametologs is inherited from a common ancestor of the closely related nightingale species. For example, nearly half of the GRC of the common nightingale consists of a large, albeit fragmented, segment homologous to the undivided segment of A-chromosome 2. This genetic material has not been identified in the western nightingale at all (Schlebusch et al., 2023).

Segmental copying of A-chromosome regions into the GRC, followed by the dispersal of these regions within the GRC, is observed in all species that have been sufficiently studied. For example, a major portion of the GRC of the zebra finch consists of dispersed sequences homologous to a segment from the short arm of chromosome 3 (Itoh et al., 2009; Torgasheva et al., 2019). The fragments from the long arm of the same chromosome and from one of the microchromosomes were found to be homologous to the GRC sequences of the siskin. The GRC of the sand martin contains material from chromosomes 4 and W, while the GRC of the great tit is partly homologous to one of the microchromosomes (Torgasheva et al., 2019).

The mechanisms of copying and dispersing fragments of A-chromosomes in the GRC remain unknown. Recombination between the GRC and A-chromosomes is unlikely A.A. Torgasheva et al. (2019, 2021) and L.P. Malinovskaya et al. (2020) did not observe in females of four studied songbird species any ectopic contacts between GRC and A-chromosomes, which could lead to recombination and/or conversion. They did not detect a self-synapsis of the GRC synaptonemal complexes, which could lead to deletions, duplications, and dispersal of sequences within the GRC, either.

The remarkable range of inter-species variability in size and heterogeneity in the genetic composition of GRC indicate an extraordinary evolutionary fluidity of this remarkable chromosome. Gametologs constantly accumulate point mutations, gradually diverging from their somatologs and gametologs of other species. At the same time, different gametologs constantly emerge within the GRCs of different species by copying from different regions of different A-chromosomes. Some gametologs amplify in the GRC, increasing its size, while the deletion of others (or the same ones?) results in its reduction. These processes of growth and contraction of the GRC affect different regions in different species, enhancing the divergence of their GRCs.

The distribution of GRC among birds (Torgasheva et al., 2019) and the estimates of the divergence time between gametologs and somatologs of the zebra finch (Biederman et al., 2018; Kinsella et al., 2019) unequivocally indicate a monophyletic origin of GRC in the songbirds.

A.A. Torgasheva et al. (2019) hypothesized that the first GRC could have arisen in the genome of ancestral songbirds through a trisomy of one of the microchromosomes. The ancient nature of GRC in songbirds and its extraordinary genetic fluidity leave no hope of finding A-chromosome paralogs, which would be the last common ancestor of GRC in all songbirds.

An analysis of B-chromosomes in different species suggests that they are derived from fragments of A-chromosomes containing the centromere, which arise during chromosomal rearrangements in the germ line (Camacho et al., 2000; Rubtsov, Borisov, 2018; Poignet et al., 2021). Interchromosomal rearrangements are fixed in bird evolution much less frequently than in other vertebrate taxa. The diploid number (2n) of the absolute majority of bird species varies within a very narrow limit: 80 ± 2, and most of the chromosomes in modern birds are syntenic to the reptilian chromosomes (Warren et al., 2010; Griffin, Burt, 2014; Damas et al., 2018). Intrachromosomal rearrangements, particularly inversions, play a significant role in the evolution of bird karyotypes, sometimes acting as one of the mechanisms of speciation (Hooper, Price, 2017; Bravo et al., 2021). Recombination within the inversion loops in heterozygotes for inversion may lead to the formation of chromosome fragments containing functional centromeres. One of such fragments could have become the ancestor of GRC in songbirds

It can be hypothesized that the proto-GRC became a chromosome restricted to germ cells at the earliest stages of its evolution. Many B-chromosomes exhibit genotaxis, accumulating in generative tissue and being deficient in somatic cells (Camacho, 2022). The evolutionary significance of this phenomenon is evident: it reduces the pressure of natural selection on genes localized on these chromosomes. However, the mechanisms underlying this phenomenon remain unknown.

A.A. Torgasheva et al. (2019) suggested that the proto- GRC might already contain multiple copies of somatic genes controlling the development and functioning of reproductive organs. Therefore, it could be selected for because it provided a higher dosage of these genes. This suggestion appears plausible given the extreme economy of the avian genome (Griffin, Burt, 2014). However, it is more likely that at the time of its origin, the GRC was a typical B chromosome, i. e., an efficient parasite that ensured its transmission without considering the host’s interests. Useful genes were probably copied into the GRC at later stages of its evolution. The few B-chromosome variants that persist in host genomes for extended periods and are widespread in populations become gradually “domesticated” acquiring properties beneficial to the hosts (Johnson Pokorná, Reifová, 2021).

The question is what these properties are. By analogy with other examples of programmed DNA elimination discussed above, it can be hypothesized that the GRC may contain unique or amplified copies of the genes controlling early development that exhibit antagonistic pleiotropy. Such genes may be necessary for early development, but their expression in later stages of life can be dangerous. In that case, the GRC can be considered the genetic equivalent of a startup disk or a boot drive. Supporting this hypothesis is the discovery of the evolutionarily ancient gametologs bicc1, trim71, and puf60, the products of which may play important roles in embryonic cell differentiation and cell cycle control (Kinsella et al., 2019).

Another class of genes that could have been evolutionarily advantageous when additional copies were created within the GRC are genes involved in the control of development and proliferation of germ cells (e. g., prdm1 and BMP15 in the zebra finch and the blue tit), as well as in the regulation of synapsis and chromosome recombination (SIX6OS1, SYCE2, SYCP1, TEX12, RNF212B in the blue tit and the nightingale) (Mueller et al., 2023).

It has been suggested that the GRC may participate in sex determination and serve as the basis for a new system (where GRC/0 = X/0 – males, GRC/GRC = X/X – females), which has already emerged or is emerging in the songbirds on top of the typical ZZ-ZW system found in all birds (Stöck et al., 2021). However, I find this proposal highly questionable. As I showed above, females with a single GRC exist and appear to develop normally, while certain males possess two GRCs in a significant portion of their germ cells.

The suppression of recombination between the GRCs, along with the extensive trafficking between the GRCs and A-chromosomes, should lead to rapid incompatibilities between the genomes of closely related species. Following the Dobzhansky–Muller model (Orr, Turelli, 2001), it can be hypothesized that within each species, the emerging GRC variants are tested for compatibility with A-chromosomes, which already exhibit considerable homology due to constant trafficking. We have already discussed that the mechanisms underlying the massive trafficking of genes from A-chromosomes to the GRC remain unknown.

Furthermore, we do not know the extent to which this trafficking is unidirectional or if there is a reverse movement from the GRC to A-chromosomes. If the trafficking occurs in both directions, the GRC may serve as a generator and incubator of new A-genes. If the trafficking is unidirectional, bird species should rapidly diverge into matrilines based on GRC composition. This could lead to genetic incompatibilities not only between individuals from geographically isolated populations but also within populations, offering broad opportunities for allopatric and sympatric speciation.

Passerines represent the most species-rich suborder of birds, encompassing 6,500 species out of the known 10,500 bird species. All passerines, and only they, possess the GRC. Could this be a contributing factor to their high species number and diversity?

## Conflict of interest

The authors declare no conflict of interest.
